# Robotic-enhanced hybrid ablation for persistent and long-standing atrial fibrillation: Early assessment of feasibility, safety, and efficacy

**DOI:** 10.1016/j.xjtc.2024.02.013

**Published:** 2024-03-02

**Authors:** Stefano Schena, Jacob Lindemann, Anne Carlson, Trisha Wilcox, James Oujiri, Marcie Berger, Mario Gasparri

**Affiliations:** a Division of Cardiothoracic Surgery, Medical College of Wisconsin, Milwaukee, Wis; b Division of Cardiology/Electrophysiology, Medical College of Wisconsin, Milwaukee, Wis

**Keywords:** atrial fibrillation, hybrid ablation, left atrial posterior wall, robotic, rhythm follow-up

## Abstract

**Objectives:**

To assess feasibility, safety, and early efficacy of robotic-enhanced epicardial ablation (RE-EA) as first stage of a hybrid approach to patients with persistent (PsAF) and long-standing atrial fibrillation (LSAF).

**Methods:**

Single-center, retrospective analysis of patients with documented PsAF and LSAF who underwent RE-EA followed by catheter-guided endocardial ablation. Postoperatively, patients were monitored for major adverse events and underwent rhythm follow-up at 3 and 12 months.

**Results:**

Between January 2021 and June 2023, we performed RE-EA in 64 patients (73.5% male, CHA_2_DS_2_-VASc 2.7 ± 1.6, BMI 34.1 ± 6.3 kg/m^2^). Mean AF preoperative duration and left atrial volume index were, respectively, 85 months and 47.5 mL/m^2^. Through the robotic approach, the intended lesion set was completed in all patients without cardiopulmonary bypass support, conversion to thoracotomy/sternotomy, blood transfusions, or perioperative mortality. The average LOS was 1.7 days, with only 1 patient requiring intensive care unit admission and >65% of patients discharged within 24 hours. At follow-up, 2 (3.1%) patients experienced new left pleural effusion or hemidiaphragm paralysis requiring treatment. There were no readmissions related to AF, stroke, thromboembolic events, or deaths. The mean interval between the epicardial and endocardial stages of the procedure was 5.9 months. Rhythm follow-up showed AF resolution in 73.4% and 71.9% of patients at 3 and 12 months, respectively.

**Conclusions:**

RE-EA is a feasible and safe, first-stage approach for the treatment of patients with PsAF and LSAF. It improves exposure of the intended targets, favors short hospital stay, and facilitates return to activity with satisfactory AF treatment in the short term.

## Graphical abstract

**Figure:**
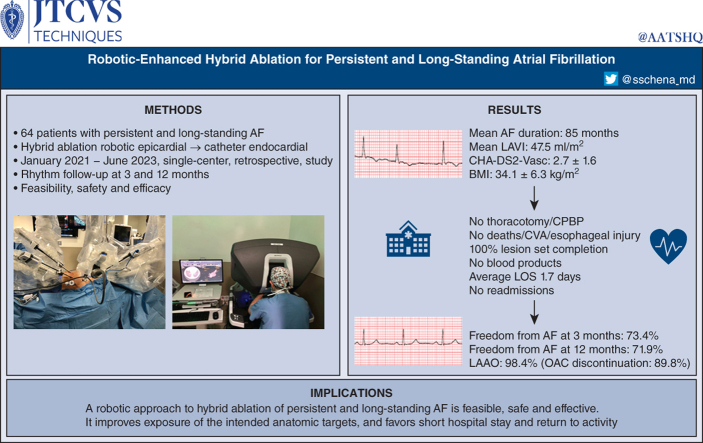

**Figure:**
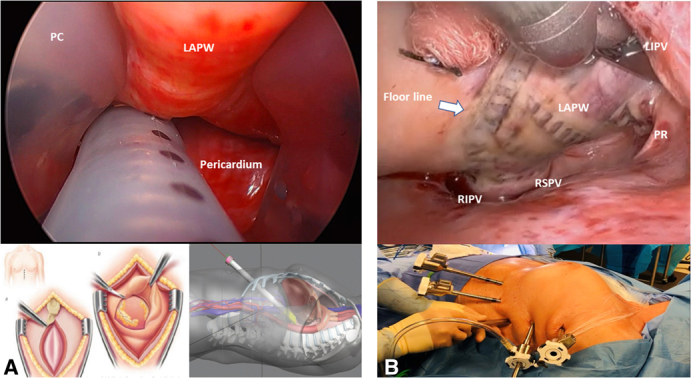
Access to left atrial posterior wall and pulmonary veins: (A) subxiphoid access versus (B) robotic view.

**Central Message**

A robotic, epicardial approach to hybrid ablation of persistent atrial fibrillation safely and effectively improves visualization and treatment of the target anatomy while promoting faster recovery.

**Perspective**

Hybrid ablation, combining a surgical epicardial and catheter endocardial approach to AF, has developed with remarkable results, impacting the need for lifelong oral anticoagulation and antiarrhythmic therapy with greater quality of life. Robotic technology may further decrease invasiveness and facilitate ablation of the intended anatomic targets while accelerating recovery and improving outcomes.

Atrial fibrillation (AF) is the most prevalent arrhythmia worldwide, with a projected impact on 15 million individuals, in the United States alone, by 2050.^1^ The effects of paroxysmal atrial fibrillation (PAF), progressing into persistent atrial fibrillation (PsAF) or long-standing atrial fibrillation (LSAF), are particularly evident in elderly patients, in whom increased risk of congestive heart failure,^2^ cognitive dysfunction,^3^ stroke,^4^ and premature death^5^ are known. Rhythm or rate control medical therapy, combined with lifelong oral anticoagulation (OAC), has shown no benefit in terms of mortality in large randomized trials.^6^^,^^7^ Although catheter ablation of non-PAF is characterized by marginal results, surgical ablation (Cox-maze procedure) remains the treatment with the most favorable outcomes.^8^ Surgical treatment of AF, however, remains underperformed.

In an effort to replicate the success of the Cox-maze while further minimizing invasiveness, hybrid ablation (HA) techniques combining surgical epicardial ablation and catheter-based endocardial ablation have evolved. The most popular of these is the Convergent procedure,^9^ a two-stage multidisciplinary approach taking advantage of (1) surgical, closed-chest, epicardial access to the left atrial posterior wall (LAPW), typically via a subxiphoid window; and (2) percutaneous, catheter-based, mapping and endocardial completion of the intended epicardial lesion set.

The introduction of a robotic surgical platform consistently facilitates cardiothoracic surgical procedures and has already proved its efficacy for left atrial appendage occlusion (LAAO).^10^ The aim of this study is to demonstrate safety, feasibility, and efficacy of a robotic-enhanced approach to hybrid ablation (RE-HA) of PsAF and LSAF.

## Methods

### Clinical Protocol and Patients

This study was reviewed by the Medical College of Wisconsin Institutional Review Board, who approved the protocol (PRO00039130, approved December 30, 2020, Figure E1). Given the observational and retrospective nature of the study, the need for informed consent was waived.

**Figure 1:**
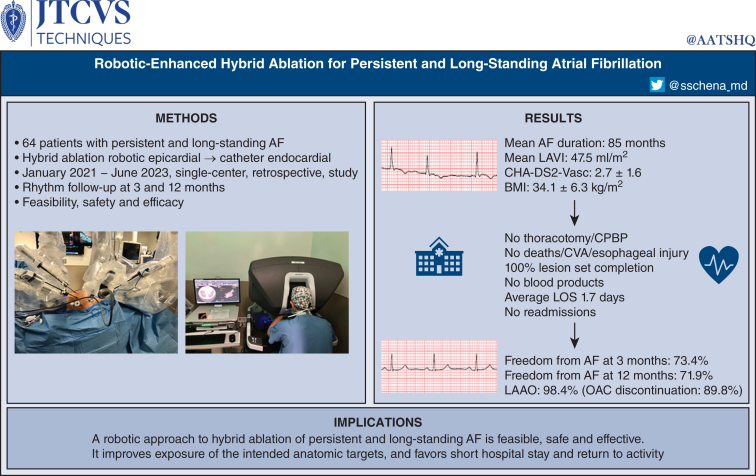
Patient cohort and features of the study. *AF*, Atrial fibrillation; *LAVI*, left atrial volume index; *CHA*_*2*_*DS*_*2*_*-VASc*, Congestive heart failure, Hypertension, Age, Diabetes, Stroke, Vascular Disease Score; *BMI*, body mass index; *CPBP*, cardiopulmonary bypass; *CVA*, cerebrovascular accident; *LOS*, length of stay; *LAAO*, left atrial appendage occlusion; *OAC*, oral anticoagulation.

This is a retrospective, single-center, analysis of safety and efficacy outcome data of 64 consecutive patients who underwent RE-HA at our institution between January 2021 and June 2023 (Figure 1). Indication for the procedure was based on the most recent Heart Rhythm Society/European Heart Rhythm Association/European Cardiac Arrhythmia Society guidelines,^11^ where *persistent* AF is defined as continuous AF that is sustained beyond 7 days and either less (“early”) or more (“true”) than 3 months in duration, whereas *long-standing* AF is defined as continuous AF greater than 12 months in duration.

All surgical, epicardial, stages of the procedure were performed by the same two surgeons (M.G. and S.S.). The endocardial catheter stage was performed by one of 6 referring EP cardiologists. All intraoperative transesophageal echocardiography (TEE) was performed by the same TEE-credentialed cardiac anesthesiology team, whereas postoperative TEE follow-up was completed by the EP cardiologists involved in the hybrid procedures. The endocardial ablation portion of the procedure was completed within 12 to 24 weeks.

### Technology

**Figure 2:**
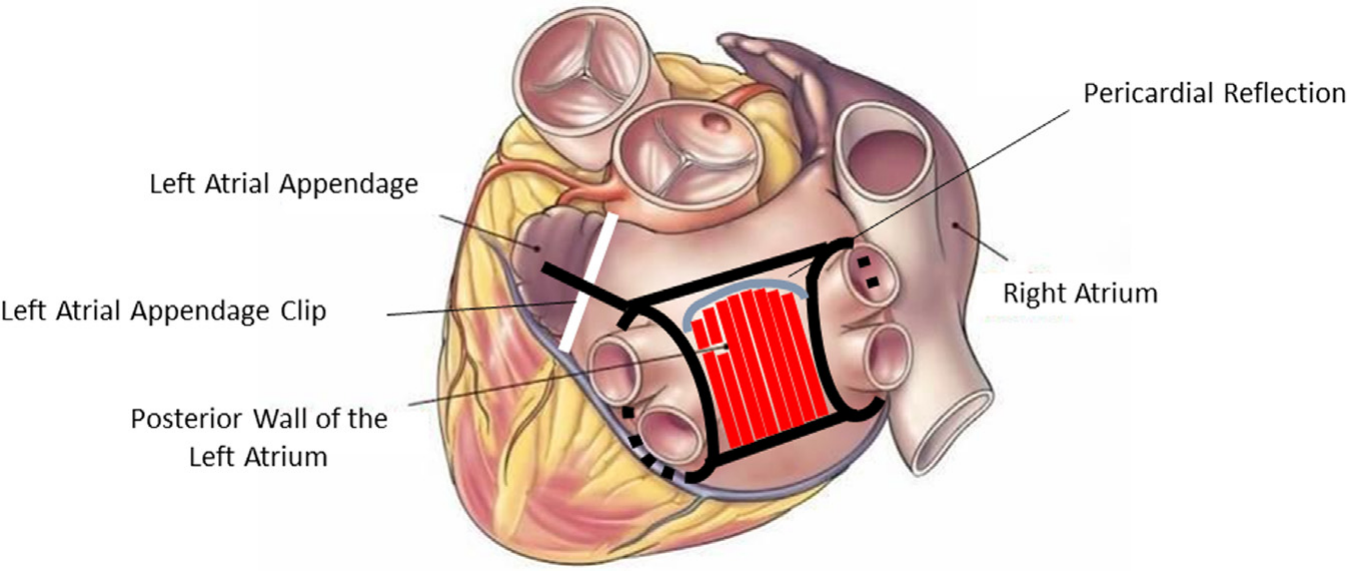
Diagram of the robotic-enhanced epicardial ablation lesion set.

Robotic epicardial ablation was performed with the Da Vinci Xi system. Intraoperative signal and impedance-guided tissue coagulation was achieved with a 3-cm, irrigated, Epi-sense unipolar probe (CDDP-4330; AtriCure, Inc). A complete epicardial lesion set (Figure 2) was defined as (1) bilateral, semicircumferential pulmonary veins (PVs) ablation; (2) complete box lesion set (connecting lesions between all PVs); (3) LAPW ablation (except for the area underneath the pericardial reflection [PR]); (4) division of the ligament of Marshall; (5) ablation of the left atrial ridge (extending from the left atrial appendage [LAA] to the left superior pulmonary vein [LSPV]); and (6) exclusion of the LAA, via epicardial clip (AtriClip PRO2 or PRO-V devices).

Each epicardial lesion was completed with as many applications as needed to reach adequate drop in tissue conductance. Confirmation of LAPW and PVs isolation was obtained in patients who were successfully electrically cardioverted to restore sinus rhythm. This was performed intraoperatively through testing of bidirectional block with the use of an isolator, long, TT transpolar pen (AtriCure, Inc) set on pacing/mapping mode. Conduction block was also inspected, confirmed, or achieved during the endocardial, catheter-based, stage performed in the electrophysiology laboratory. After the first 9 cases combining unilateral robotic with subxiphoid epicardial access, we modified the surgical approach to a total robotic procedure without subxiphoid incision.

Finally, the LAA was considered excluded if intraoperative TEE demonstrated no persistent intraluminal flow and a residual stump of <10 mm, as previously described.^12^ Findings would then be confirmed by TEE reassessment at the time of the endocardial, catheter-based, stage and/or by gated cardiac computed tomography angiography (CTA).

### Preoperative Evaluation and Data Collection

All patients with PsAF and LSAF received full preoperative assessment and underwent transthoracic echocardiography as well as CTA at Froedtert Hospital/Medical College of Wisconsin. Relevant demographics, baseline characteristics, and risk-stratification data were collected in an Excel (Microsoft Corp) institutional-protected database. Aside from the conduct of the procedure (robotic vs subxiphoid), all patients who underwent RE-HA were otherwise selected, managed, and followed as per our institutional standard for patients treated with a hybrid epicardial/endocardial approach.

Outcome and follow-up data were obtained from longitudinal outpatient follow-up visits, electronic medical record review, phone interviews, rhythm data transmission from previously implanted devices (implantable cardioverter defibrillator, pacemakers, or loop recorders), and reports of wearable heart rhythm monitors (7-day Holter) scheduled per protocol.

### Surgical Technique

All procedures were performed via a unilateral, left-sided, approach (Video 1) with the patient under general anesthesia with double-lumen endotracheal intubation for selective right lung ventilation, and placement of an esophageal temperature probe to constantly monitor for possible retrocardiac thermal spreading. A thorough TEE evaluation was performed in every patient to confirm the absence of intracardiac thrombi.

**Figure 3:**
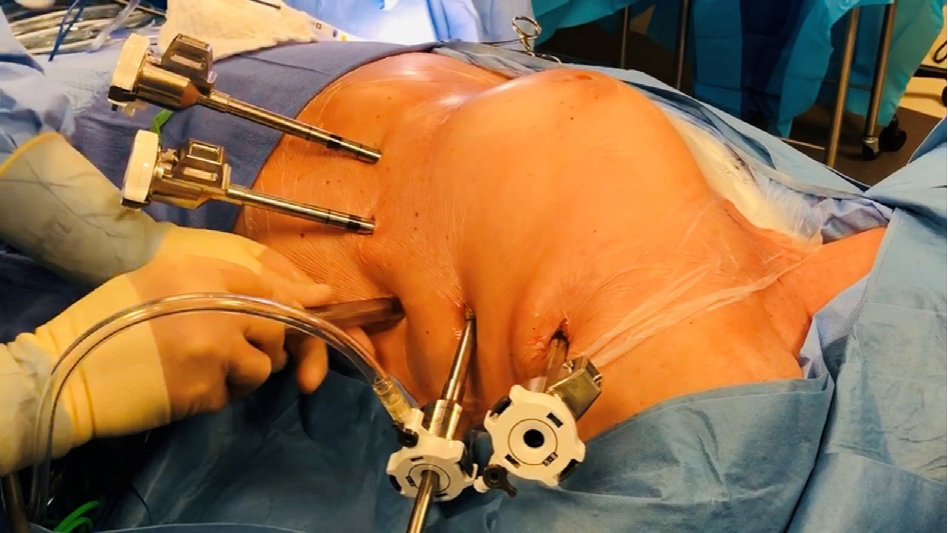
Robotic port placement on the left hemithorax.

**Figure E2:**
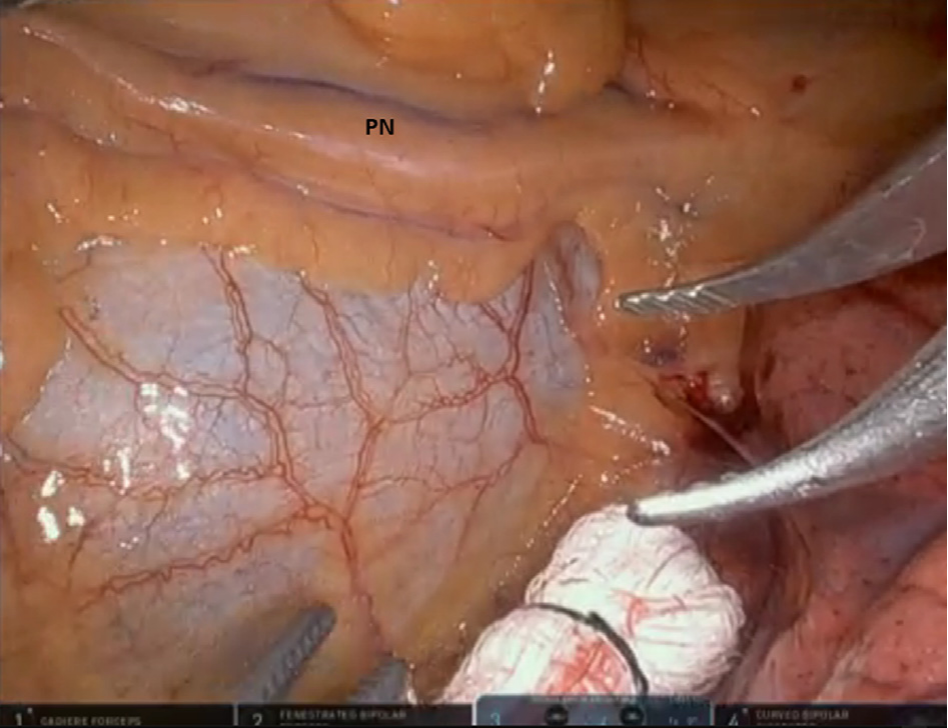
Robotic view of the left lateral aspect of the pericardium and phrenic nerve (*PN*).

**Figure E3:**
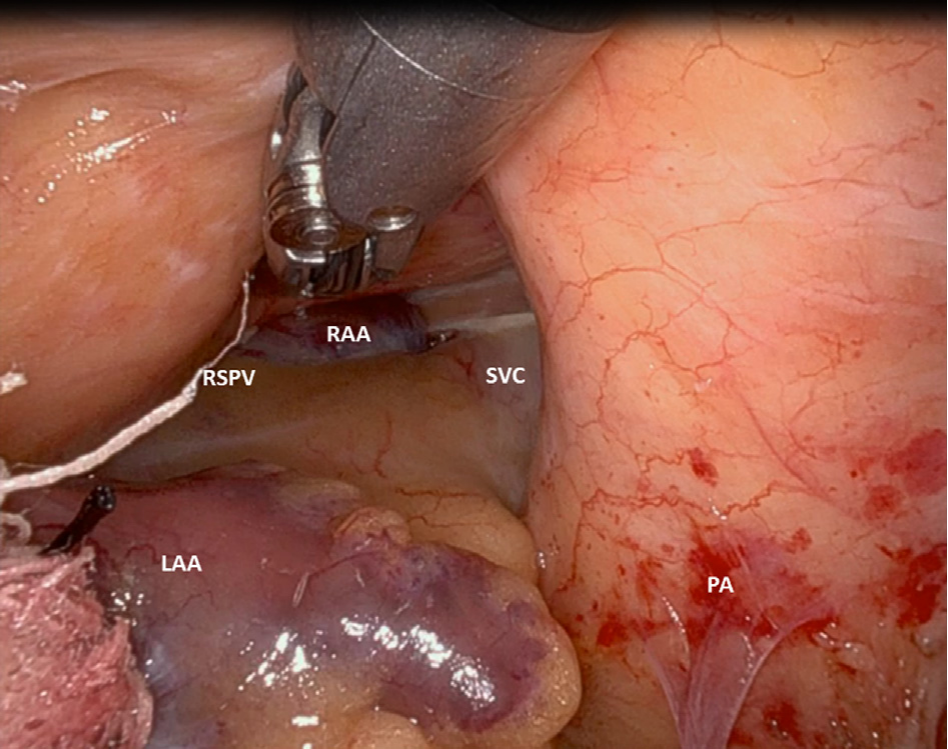
Robotic magnification of the sinus transversus, after division of the ligament of Marshall, with optimized view of the roof of the left atrium (R), right atrial appendage (*RAA*), superior vena cava (*SVC*), and left atrial appendage (*LAA*). *RSPV*, Right superior pulmonary vein; *PA*, pulmonary artery.

**Figure E4:**
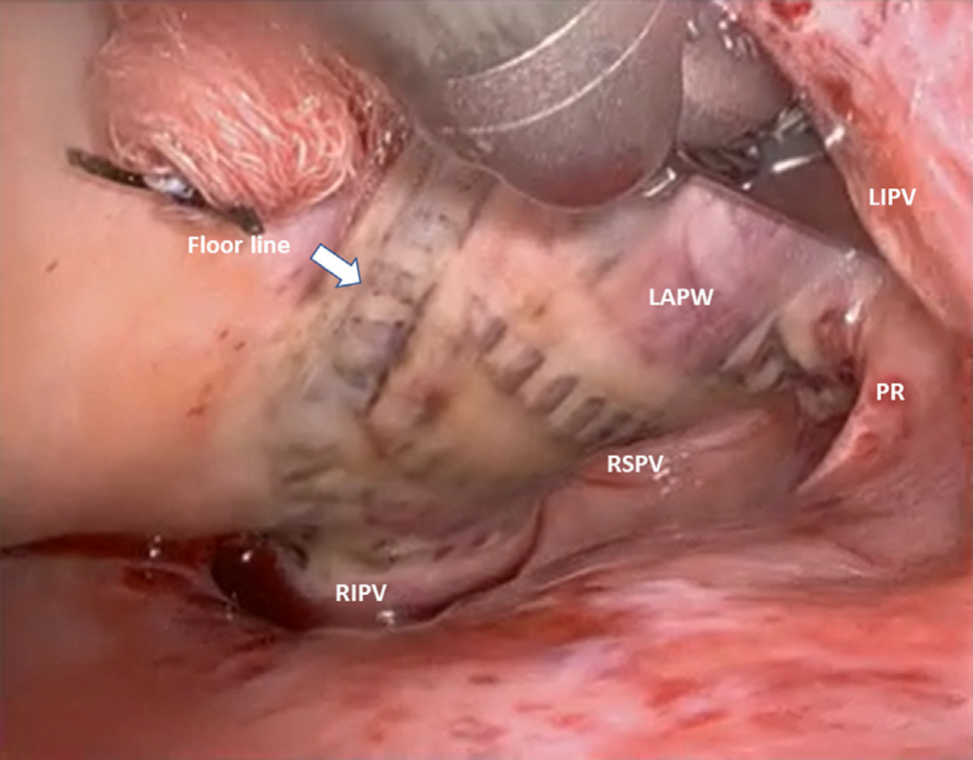
Robotic magnification of the left atrial posterior wall (*LAPW*) with optimized view of the right inferior pulmonary vein (*RIPV*), right superior pulmonary vein (*RSVP*), pericardial reflection (*PR*), left inferior pulmonary vein (*LIPV*) and “floor” line.

The patient is placed supine and the left chest slightly elevated with a roll placed cephalad-to-caudad, just below the shoulder. The left arm is slightly flexed at the elbow and padded on an armboard, below the left posterior axillary line. The left lung is deflated, and 4 robotic ports are inserted in “hockey-stick” fashion (Figure 3). The camera is inserted through an 8-mm port placed first in the mid- to anterior axillary line midway between the xiphoid and the sternal notch. Carbon dioxide insufflation at 8 mm Hg pressure is used. The remaining ports are then placed under camera visualization with manual use of the 0-degree robotic scope. The use of long trocars with maximized clearance on the robotic arms is critical, as this allows best separation of these arms and avoids collisions. The upper port is placed in correspondence of the LSPV, in the anterior axillary line, which usually corresponds to the third or fourth intercostal space (ICS). Two more long, 8-mm ports are then placed respectively at the level of the cardiophrenic angle, in the midclavicular line and anterior axillary line, usually at the seventh or eighth ICS. Finally, a 12-mm AirSeal port (ConMed Corp) is placed in the sixth ICS at the level of the midaxillary line and serves as the bedside assistant port through which the LAA clip is ultimately placed. The robotic platform is docked and the left pleural cavity is inspected. Any adhesions are taken down and the lung allowed to retract posteriorly. The procedure is performed on a beating heart and does not require systemic heparinization or cardiopulmonary bypass. The left lateral aspect of the pericardium is then visualized, the phrenic nerve identified, and the pericardium entered 1 cm posteriorly (Figure E2). A longitudinal pericardiotomy is created in order fully expose the LAA, left PVs, pulmonary artery, and obtuse margin. The anterior edge of the pericardiotomy is retracted superiorly with a braided suture externalized through the chest wall. The ligament of Marshall is identified and divided. The transverse sinus is then entered and the roof of the left atrium visualized all the way to the right superior pulmonary vein (RSPV). The superior vena cava and right atrial appendage are also identified (Figure E3). The PR around left and right PVs is developed with blunt dissection. The 3-cm Epi-sense unipolar probe is then inserted through the 12-mm AirSeal port and advanced within the transverse sinus to the confluence of the RSPV and left atrium. A “roof” ablation line is initiated at this level and extended proximally to the origin of the LSPV. Each targeted area is ablated at least twice based on measured tissue impedance and probe-detected electrical silence. The probe is then positioned on the antrum of the left PVs and an ablation line connecting the 2 vessels anteriorly is performed. The posterior pericardial space is then dissected and the unipolar probe positioned accordingly in order to perform semicircumferential isolation of both the left PVs. The heart is then retracted anteriorly and the LAPW inspected identifying both the right and left PVs, as well as the PR. The probe is then used to achieve semicircumferential ablation of the right PVs. An ablation line is then created by connecting the inferior aspect of the right inferior pulmonary vein to the inferior aspect of the left inferior pulmonary vein (“floor line”) and the entire LAPW is ablated proceeding, in a parallel fashion, from the right towards the left PVs and from floor line to the PR (Figure E4). Although we continuously monitor the esophageal temperature throughout the procedure, esophageal heating is rarely an issue, as the heart is elevated, which minimizes thermal spreading. Once the LAPW is completed, we allow the heart to fall back to its normal anatomic position and return our attention to the LAA. One last ablation is performed to address the left atrial ridge. Next, the LAA is sized at its base and occluded using an AtriClip, as previously described.^10^ We then undock the robot and remove all instruments, insert a 19-French Blake drain through one of the port sites, and close all incisions. All patients are awakened from general anesthesia and extubated in the operating room before being transferred to the recovery room.

### Endocardial Ablation

**Figure 4:**
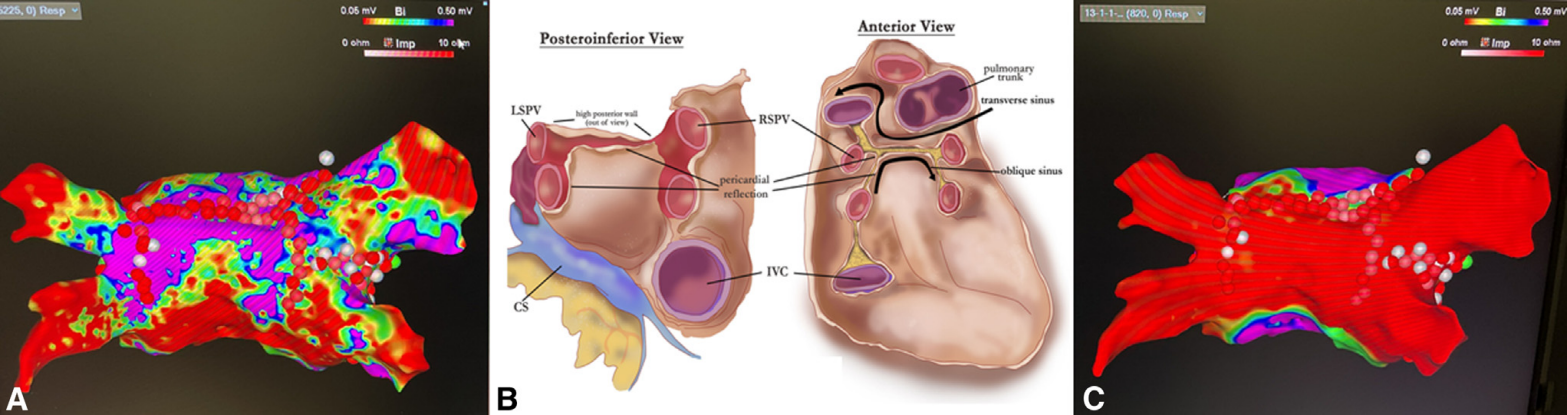
Electrocardiographic mapping showing extent of epicardial ablation (A, in *red*) progressively tapering into the pericardial reflections (*purple*), and endocardial, catheter-based, completed ablation of the LAPW and PVs (C). Diagram of posterior pericardial reflections (B, courtesy of John-Ross D. Clarke, MD).

At the time of endocardial ablation, patients underwent 3-dimensional CARTO (Biosense Webster, Inc) endocardial mapping (Figure 4, *A*) followed by a standard ablation set including a roof line, LAPW between LSPV and RSPV, completion wide-area circumferential ablation for PVs isolation and, although rarely required following the epicardial stage, touch-up ablation of the inferior border of the LAPW between the inferior PVs (Figure 4, *C*). If typical or atypical flutter was also identified, patients had ablation lines to the mitral valve isthmus, between left inferior pulmonary vein and the mitral valve annulus, or at the cavotricuspid isthmus at approximately 6-o'clock position between the tricuspid valve and the inferior vena cava.

### Follow-up Protocol

Patients underwent telemetry monitoring after completion of each stage of the procedure. They also underwent both outpatient follow-up within 14 days of discharge as well as rhythm surveillance. The latter was performed at 3 and 12 months from the completion of the endocardial stage through (1) intermittent 12-lead electrocardiograms obtained at each visit; (2) wearable 7-day Holter monitor; and (3) rhythm analysis of pre-existing automated implantable cardioverter-defibrillator, pacemakers, or loop recorders. In addition, rhythm data were also retrospectively harvested for all readmissions and emergency department visits related to a diagnosis of “arrhythmia.”

### Statistical Analysis

Continuous variables were expressed as mean with standard deviation, whereas categorical variables were calculated as percentages of absolute numbers, using a standard Excel (Microsoft 365; Microsoft Corp) statistical package. Values were rounded to the first decimal.

A failure of the HA procedure was defined as any episode of AF, flutter, or atrial tachycardia longer than 30 seconds detected after a 3-month blanking period and at 12-month follow-up during continuous hearth rhythm monitoring tests obtained after discontinuation of class I/III antiarrhythmic drugs (AADs).^11^ A failed epicardial LAA exclusion was defined as any appendage with either a stump >10 mm or residual flow (detected by TEE) or contrast enhancement (by CTA) at follow-up.

## Results

Between January 2021 and June 2023, a total of 64 RE-HA procedures were completed. All patients were treated according to protocol, and each procedure was considered completed when both the robotic epicardial and the transcatheter endocardial ablations were performed in sequential fashion. Of the 64 patients treated, 37 underwent synchronized cardioversion of their pre-existing AF after anesthesia induction. Only the first 9 patients in the series underwent access to the LAPW through a subxiphoid pericardial incision.

### Patient Characteristics

Preoperative baseline characteristics and clinical data are summarized in Table 1. More than 67% of patients had obesity (BMI ≥30 kg/m^2^), with 21 (48.8%) and 9 (20.9%) being, respectively, class II (BMI 35-39) and class III (BMI ≥40) obese. Approximately 65% of patients were symptomatic with mild exertion ( NYHA class II). None of the patients presented with significant, concurrent mitral, tricuspid, or aortic valvular disease. Mean AF duration before surgery was 85 months and, in 70.3% of cases, this was persistent in nature. Fourth-eight patients (75%) had 1 or more failed cardioversions as part of their AF history. Medical management consisted of class I/III antiarrhythmic in 65.6% and oral anticoagulation in 93.7% of cases.

**Table 1:** Baseline characteristics of patients who received RE-HA (N = 64)

Age, y	64.5 ± 9
Male sex	47 (73.4%)
BMI, kg/m^2^	34.1 ± 6.3
NYHA class	
I	5 (7.8%)
II	42 (65.6%)
III	17 (26.5%)
Arrhythmia history	
AF duration, mo	85 ± 68.5
AF type	
Paroxysmal	4 (6.2%)
Persistent	45 (70.3%)
Long-standing	15 (23.4%)
Previous cardioversion	48 (75%)
CHA_2_DS_2_-VASc	2.7 ± 1.6
HAS-BLED	3 ± 1.4
Medical history	
Hypertension	50 (78.1%)
Diabetes	20 (31.2%)
Dyslipidemia	32 (50%)
Obesity	43 (67.2%)
Thyroid disorders	12 (18.7%)
Obstructive sleep apnea	29 (45.3%)
Anxiety disorder	10 (15.6%)
Stroke/TIA	7 (10.9%)
Chronic kidney disease	7 (10.9%)
HFrEF	9 (14%)
Coronary artery disease	12 (18.7%)
COPD	5 (7.8%)
Preoperative management of AF	
Class I/III antiarrhythmic drug	42 (65.6%)
Oral anticoagulant	60 (93.7%)
Preoperative echocardiogram	
LV ejection fraction, %	54.6 ± 10.3
LA volume, mL	104.5 ± 41.3
LA volume index, mL/m^2^	47.5 ± 18
Previous PM/ICD/CRT implantation	12 (18.7%)
Previous sternotomy	3 (4.7%)

*BMI*, Body mass index; *NYHA*, New York Heart Association; *AF*, atrial fibrillation; *CHA*_*2*_*DS*_*2*_*-VASc*, Congestive heart failure, Hypertension, Age, Diabetes, Stroke, Vascular Disease Score; *HAS-BLED*, Hypertension, Abnormal renal/liver function, Stroke, Bleeding history or predisposition, Labile INR, Elderly, Drugs/alcohol concomitantly; *TIA*, transient ischemic attack; *HFrEF*, heart failure with reduced ejection fraction; *COPD*, chronic obstructive pulmonary disease; *AF*, atrial fibrillation; *LV*, left ventricular; *LA*, left atrial; *PM/ICD/CRT*, pacemaker/implantable cardioverter defibrillator/cardiac resynchronization therapy.

At preoperative echocardiographic evaluation, the mean ejection fraction was 54.6%. Not unexpectedly, the patients in this cohort presented with large left atria (mean left atrial volume index 47.5 mL/m^2^), with 24 (37.5%) of them being considered severely enlarged (>48 mL/m^2^) per previously delineated criteria.^13^ The mean CHA_2_DS_2_-VASc was 2.7, and HAS-BLED was 3. Finally, 12 (18.8%) patients had a previously implanted rhythm-control device whereas 3 (4.7%) patients had previous cardiac surgery through sternotomy.

Observed intraoperative features are described in Table 2. Of the 3 patients with pre-existing sternotomy, 2 had previous coronary artery bypass grafting and 1 had septal myectomy. In addition, unexpected pericardial adhesions were identified in 4 other cases and successfully managed with the robotic approach. The epicardial ablation set could be successfully completed in all patients. One patient did not undergo LAAO as the result of very dense adhesions that did not allow for safe definition of its margins. The patient subsequently underwent successful, percutaneous, placement of a WATCHMAN device (Boston Scientific). No adverse events occurred intraoperatively that led to conversion of the robotic approach to either thoracotomy or sternotomy, blood products transfusion, emergent institution of cardiopulmonary bypass, or abandonment of the procedure. All patients were successfully extubated in the operating room and admitted to a regular surgical floor. In one case, temporary intensive care unit (ICU) admission was indicated because of the patient's known history of chronic congestive heart failure and need for weaning of intravenous inotropic support. The mean length of stay (LOS) was 1.7 days, whereas the mean interval between the 2 stages of the HA procedure was 5.9 months.

**Table 2:** Procedural features (N = 64)

Time between stages, mo	5.9 ± 4.1
Epicardial procedure completion	
Lesion set	64 (100%)
LAAO	63 (98.4%)
Aborted	0
Conversion to thoracotomy/sternotomy	0
Cardiopulmonary bypass requirement	0
Blood products transfusion	0
Admission to ICU	1 (1.5%)
Length of stay, d	1.7 ± 1.3

*LAAO*, Left atrial appendage occlusion; *ICU*, intensive care unit.

### Operative Morbidity and Mortality (≤30 Days)

Postoperative complications are listed in Table 3. There were no operative deaths, stroke, or esophageal injuries. No patient required temporary or permanent pacemaker insertion postoperatively. Three patients (4.7%) developed new pleural effusions and required thoracentesis. In 2 cases (3.1%), we observed left hemidiaphragm paralysis. The first resolved spontaneously with observation, whereas the second required thoracoscopic diaphragmatic plication. Lastly, during the early stages of our experience, we observed 2 (3.1%) subxiphoid incisional hernias that required repair.

**Table 3:** Postoperative complications (N = 64)

Death (at 30 d)	0
Atrioesophageal fistulas	0
Costochondral fracture	1 (1.5%)
Hemidiaphragm paralysis	2 (3.1%)
Pleural effusion/thoracentesis	2 (3.1%)
Pacemaker Insertion (at 30 d)	0
Subcutaneous emphysema	1 (1.5%)
Stroke/TIA	0
Peripheral thromboembolism	0
Wound complication	2 (3.1%)

*TIA*, Transient ischemic attack.

### Follow-up

Imaging and clinical aspects observed during follow-up are reported in Table 4. To date, no deaths have been observed during follow-up. Four (6.3%) patients have been readmitted for reasons related to AF and all during the 90-day blanking period. Four (7.7%) patients, among those who didn't already have one, required postoperative pacemaker or implantable cardioverter defibrillator implantation for indications unrelated to AF (progression of pre-existing sick sinus syndrome and nonischemic cardiomyopathy). No cerebral or peripheral thromboembolic events have occurred.

**Table 4:** Follow-up

Mean follow-up, mo (range)	11.4 (1-27)
Type of follow-up	
Serial ECG/clinical	12 (18.7%)
PM/ICD/CRT interrogation	14 (21.9%)
Loop recorder	7 (11%)
7-d Holter	31 (48.4%)
Readmission (AF related)	4 (6.3%)
LAA exclusion (at 3 mo)	62/63 (98.4%)
Pacemaker insertion	4 (7.7%)
Mortality	
AF related	0
Non-AF related	0
Follow-up	
3 mo	64/64 (100%)
12 mo	32/64 (50%)
Freedom from AF at 3 mo	47/64 (73.4%)
Freedom from AF at 12 mo	23/32 (71.9%)
OAC discontinuation at 12 mo	53/59 (89.8%)

*ECG*, Electrocardiogram; *PM/ICD/CRT*, pacemaker/implantable cardioverter defibrillator/cardiac resynchronization therapy; *AF*, atrial fibrillation; *LAA*, left atrial appendage; *OAC*, oral anticoagulation.

Proper exclusion of the LAA was appreciated at TEE performed during the endocardial catheter ablation stage. This was also confirmed in all but 1 (1.6%) patient through CTA obtained at 3 months.

Per intention-to-treat protocol, all patients were also scheduled for rhythm follow-up at 3 and 12 months. In more than 80% of cases, rhythm surveillance was obtained through wearable continuous ambulatory or interrogation of previously implanted devices. Thirty-two (50%) patients have reached the 12-month follow-up time point thus far. At completion of the estimated blanking period, 47 (73.4%) patients were free from any supraventricular tachyarrhythmia lasting more than 30 seconds. Of those 32 patients who reached the 12-month follow-up, 23 (71.9%) were free from any supraventricular tachyarrhythmias per HRS guidelines. Within this subgroup, 17 (73.4%) already had class I/III AADs discontinued at the time of rhythm monitoring.

## Discussion

This study describes a single-institution experience focused on feasibility, safety, and efficacy of RE-HA as part of a two-staged hybrid treatment of PsAF and LSAF. To our knowledge, this is the first report of such technique and the largest series with short-term follow-up.

Although the Cox-maze procedure stands as the surgical gold standard with nearly 3 decades of excellent outcomes,^14^ its use requires cardiopulmonary bypass and cardiac arrest even when performed in a minimally invasive fashion. Moreover, despite defined practice guidelines for surgical treatment of AF,^15^ concomitant ablation is performed in <25% of patients with AF undergoing surgery for other indications.^16^ In the meantime, the past decade has witnessed the introduction and growing popularity of combined, minimally invasive surgical/epicardial and percutaneous catheter/endocardial, hybrid approaches to PsAF. The minimally invasive nature of the surgical stage aims at enhancing patient recovery, whereas the endocardial catheter stage offers the possibility to achieve and confirm a transmural lesion set, as well as ablating structures not accessible epicardially (ie, cavotricuspid or mitral isthmus lines).

This approach, supported by the results of the randomized controlled CONVERGE trial,^17^ raised interest in both the cardiology and surgical communities with increasing adoption even through slightly different iterations, including LAA and PVs management,^18^ in most centers. These last 2 features have been accomplished by combining either uni- or bilateral video-assisted thoracoscopy to a subxiphoid pericardial access for the LAPW.

Understanding the rationale behind HA of AF requires recognizing how each anatomic structure contributes to the evolution of AF and can be differently affected by epicardial and/or endocardial treatment. Although the role of PVs and their antral confluence as the primary factor in onset and maintenance of PAF is clearly established, as evidenced by the efficacy of PVs catheter ablation,^19^ intervention limited to such targets has demonstrated suboptimal outcomes for PsAF.^20^ There is increasing evidence that the pathogenesis of PsAF and LSAF may be linked to the LAPW, as this is the site at which chronic changes preferentially occur. Progressive fibrosis and varying fiber orientation in this area lead to heterogenous conduction velocity, functional block and anisotropy that, when coupled with shorter refractory periods, favors local re-entry.^21^ In addition, the LAPW is in close proximity to the anterior wall of the esophagus and is traversed by a PR connecting the left and right superior PVs separating the transverse from the oblique sinuses. These anatomical features can limit both the endocardial approach, because of elevated risk of esophageal thermal spreading, as well as the epicardial approach, because of restricted access to the superior and lateral aspects of the LAPW. The combination of epicardial and endocardial ablation has therefore been found to be complementary, helping overcome certain anatomical constraints (Figure 4, *B*). Unfortunately, despite the success of the subxiphoid epicardial approach, limitations remain, including suboptimal exposure of the intended anatomic structures, ability to safely complete the procedure, and options to extend the ablation lesion set.^22^

When we embarked on our minimally invasive HA program, the robotic platform was used primarily to facilitate LAAO via AtriClip placement. The robotic technology, based on our institutional expertise and asset availability, allowed us to subsequently include the entire epicardial ablation lesion set exclusively through the left chest. In time, we have found that such approach offers several potential advantages compared with others, including subxiphoid pericardioscopy or standard video-assisted thoracoscopy.

**Figure E5:**
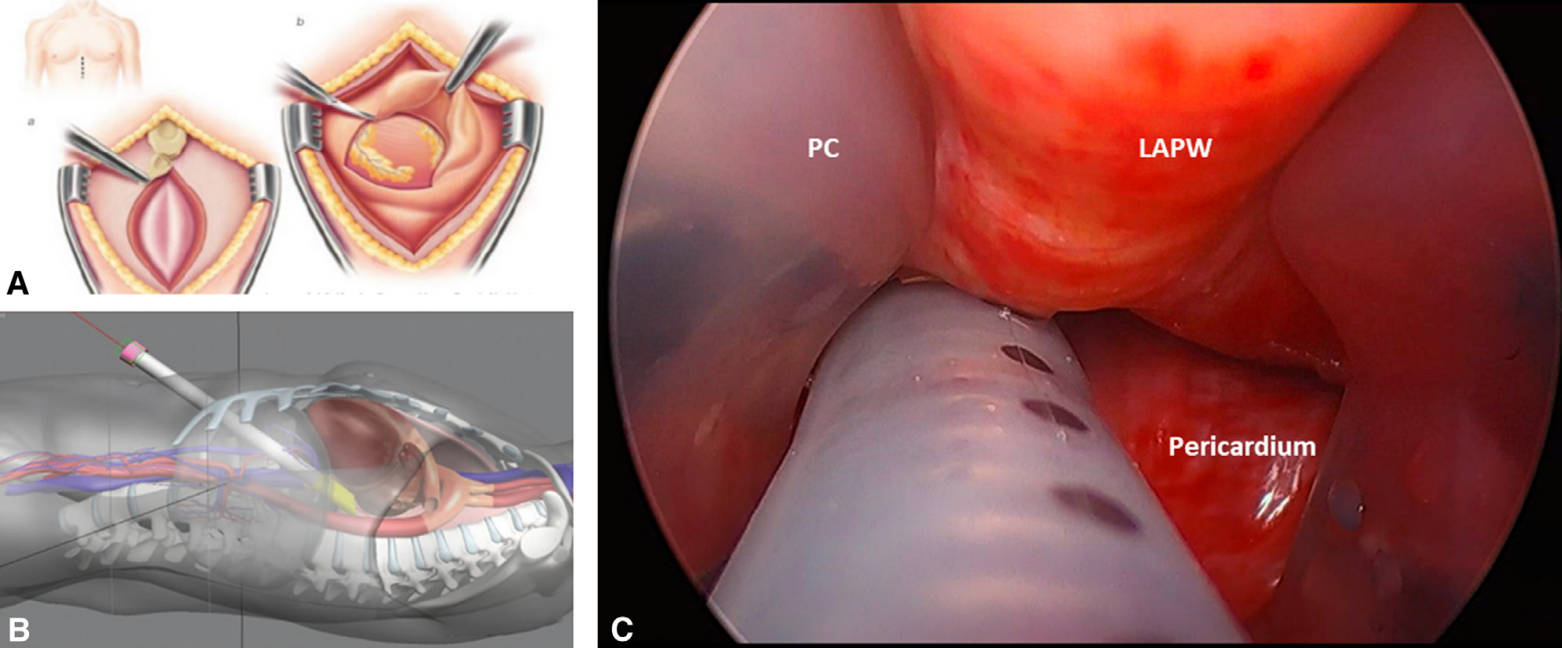
Diagram of the subxiphoid incision and pericardial access to the LAPW (A), trajectory of the pericardioscopic cannula in the mediastinum (B), view of the LAPW through the pericardioscopic cannula (*PC*). *LAPW*, Left atrial posterior wall.

First, its feasibility is entirely independent from body habitus and size. When using the subxiphoid approach, we found that with larger patients the insertion of a pericardioscopic cannula and the advancement of the ablating probe were challenging due to forced angulation required to adequately perform the procedure (Figure E5, *B*). When considering the average BMI in our cohort, the evolution toward a unilateral, left robotic approach eliminated this difficulty. In addition, especially in larger patients, a subxiphoid incision is not free of complications and indeed in our first 9 procedures using subxiphoid, pericardioscopic access to the LAPW, two patients experienced wound complications, including incisional hernia. We feel the most effective way to eliminate this complication is to avoid a subxiphoid incision altogether.

Second, the exposure offered by a lateral robotic approach is superior and decreases the frequency of esophageal heating. The insertion of a subxiphoid pericardioscopic cannula with its intrinsic “tunnel” view (Figure E5, *C*) appeared to cause a rather limited excursion of the ablating probe, hindering its precise placement, with frequent overheating of the esophagus, as well as need for frequent repositioning of both thoracoscopic camera and probe. As previously reported,^23^ the mid-lower aspect of the LAPW has an average distance from the anterior wall of the esophagus of just 2.6 mm and extends downward for several centimeters, representing a limiting factor. The robotic-assisted approach takes advantage of its lowermost port to gradually retract the inferoposterior aspect of the heart upward, hence increasing the distance between LAPW and the retropericardial structures with a much-improved exposure that is hemodynamically well tolerated. The increased distance between LAPW and esophagus is protective against its overheating. Very rarely does the esophageal temperature require cessation or pauses of the ablation, therefore leading to a more efficient process. Prominent curvature of the thoracic spine, large-sized left atria, and preoperative severity of ventricular dysfunction proved not to affect either the flow of the procedure or completion of the lesion set.

Third, we opted for a single, left-sided, access, as previous reports have also suggested that the unilateral approach not only has no appreciable detrimental effects on the overall success of the hybrid procedure but may also be associated with increased pulmonary tolerance and a significantly shorter LOS.^24^ Similarly, epicardial lesions such as superior to inferior vena cava linear ablation typically achieved through a right lateral approach do not significantly affect recurrence of atrial arrhythmias in the short term and are best addressed through a cavotricuspid flatter line ablation performed at the time of endocardial completion.^22^ Moreover, camera tridimensional magnification and intrathoracic 360° robotic instruments articulation allow for decreased rib traumatic injury, more precise identification and dissection of targeted anatomic structures, as well as safer navigation through pericardial adhesions of either inflammatory or post-sternotomy origin, as we found in 7 of our patients. These cases would presumably have been aborted if attempted via a subxiphoid approach and, although they may have been completed using standard thoracoscopic instrumentation, this would have been a significant challenge.

The decreased invasiveness offered by robotic surgery has a profound effect on postoperative care. No blood products were administered and, except for 1 patient affected by chronic congestive heart failure and requiring weaning from pharmacologic support in the ICU, all others recovered effectively on the regular floor, with an average LOS of 1.7 days. At postoperative follow-up, patients reported minimal left-sided discomfort and exhibited faster resumption of daily life activities.

One final technical consideration is that the proposed technique was developed using robotic instruments and epicardial ablation tools designed for very different purposes. Should this approach confirm its promising initial results and tools be developed specifically for AF treatment, it is foreseeable that the procedure may be further streamlined, thus becoming widely reproducible. This may, in turn, allow such a technique to be applied to an even greater number of patients with AF, a population that is currently undertreated. Nonetheless, incorporating such an approach into a clinical practice may require basic robotic thoracic skills with proper initial patient selection based on favorable anatomy and minimal comorbidities, as well as 5 proctored cases.

Although all the aforementioned advantages are beneficial, the use of the robotic platform for HA would be unsatisfactory if not associated with promising results as far as treatment of AF. In our series, 47 (73.4%) patients maintained a sinus rhythm at 3 months from completion of both stages. After this time point, 23 patients previously on class I/III AADs had these discontinued. Half of our cohort reached the 12-month rhythm follow-up since implementation of our hybrid approach, and the observed trend toward freedom from supraventricular tachyarrhythmias remained consistent among all those patients in whom class I/III AADs had been discontinued before this point in time (17 of 23, 73.9%). Cardiac CTA and/or TEE demonstrated effective LAAO in all (98.4%) but 1 patient, confirming the reported feasibility of this critical step of AF management when performed with robotic technology.^10^ This led to discontinuation of oral anticoagulation in 53 (89.8%) of patients.

The presented approach appears to also seamlessly favor bidirectional block testing by means of either commercially available pacing/sensing pens or more sophisticated, high-density, epicardial mapping systems when available. Validation of epicardial ablation during hybrid treatment of AF has been questioned in the past because of the possibility of false-positive results related to tissue edema. Such assumption, however, has been challenged and even in unilateral left-sided thoracoscopy where the RSPV can be difficult to reach, epicardial bidirectional conduction block appears to correlate well with findings obtained through endocardial testing.^25^

In terms of clinical efficacy, our results are in line with contemporary, larger, series and systematic meta-analyses using a similar unilateral approach^26^, ^27^, ^28^, ^29^ where freedom from AF without AAD ranges between 65% and 80%. We observed lower rates of perioperative complications, ICU requirement, LOS, and readmission rate that could make the robotic approach more appealing to patients and further enhance multidisciplinary approach to AF treatment.

### Limitations

This study presents several limitations. It is a single-center, retrospective study conducted at a tertiary center that represents a statewide referral for management of patients with all types of atrial tachy-arrhythmias. The institution routinely manages a high volume of robotically assisted cardiothoracic surgery cases, and the authors are surgeons with advanced robotic skills, which may not reflect the general practice. In addition, the patient sample size offers difficulties in terms of adjusting for baseline differences and generalization of the results in the long-term. This is in part also caused by factors such as surgeons' experience, performance of the early cases with a partially different surgical approach (ie, subxiphoid incision), and lack of reliable rhythm follow-up in 18% of patients (ie, continuous electrocardiogram rather than wearable monitors due to restrictions related to COVID-19 pandemic). Studies comparing robotic with other thoracoscopic approaches for the epicardial stage of hybrid AF ablation, including longer follow-up, will be necessary. Finally, the feasibility of this approach in patients with previous sternotomy will require evaluation in a larger cohort.

## Conclusions

Hybrid management of PsAF or LSAF in which a robotic-enhanced approach is used for the epicardial ablation stage is feasible, safe, and effective in the short term. The unique technical advantages offered by robotic surgery also favor shorter LOS and prompt recovery.

## Conflict of Interest Statement

S.S., J.O., and M.G. serve as AtriCure consultants (no honorary fees). All other authors reported no conflicts of interest.

The *Journal* policy requires editors and reviewers to disclose conflicts of interest and to decline handling or reviewing manuscripts for which they may have a conflict of interest. The editors and reviewers of this article have no conflicts of interest.

## Figures and Tables

**Figure E1:**
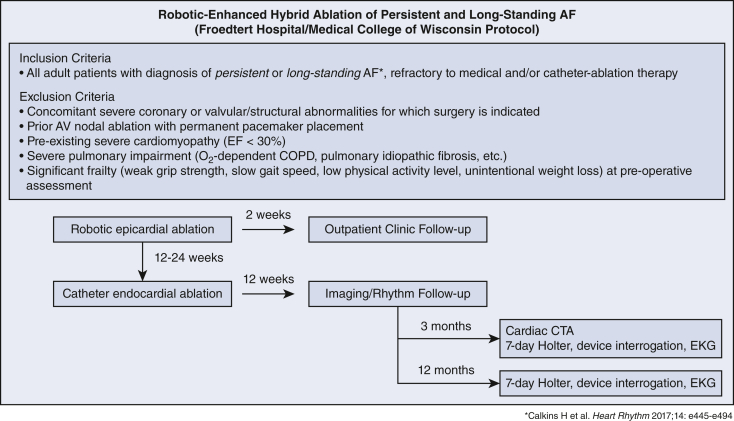
Institutional protocol for treatment and follow-up of patients with PsAF and LSAF undergoing RE-HA.

## Supplementary Data

Video 1 Robotic-enhanced hybrid ablation for PsAF and LSAF. Procedure steps. Video available at: https://www.jtcvs.org/article/S2666-2507(24)00069-5/fulltext.
