# Supplementary figures and images for: Robotic-enhanced hybrid ablation for persistent and long-standing atrial fibrillation: Early assessment of feasibility, safety, and efficacy
Source: JTCVS Tech. 2024 Mar 2;25:81–93. doi: 10.1016/j.xjtc.2024.02.013 (PMC11184487; doi:10.1016/j.xjtc.2024.02.013)

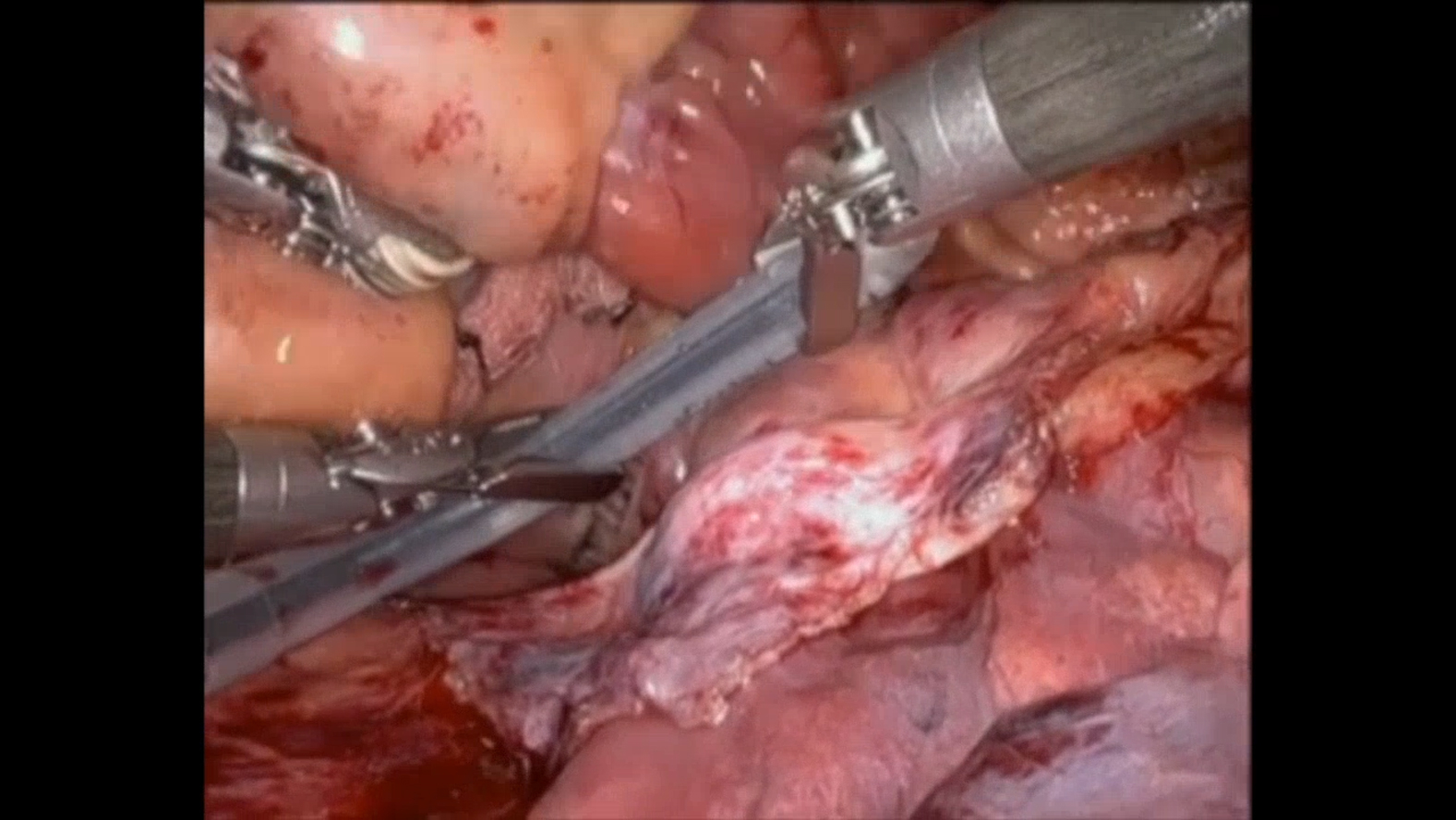

Supplement: Video 1 — Robotic-enhanced hybrid ablation for PsAF and LSAF. Procedure steps. Video available at: https://www.jtcvs.org/article/S2666-2507(24)00069-5/fulltext. [file fx2.jpg]
